# A histomorphometric study to evaluate the therapeutic effects of biosynthesized silver nanoparticles on the kidneys infected with *Plasmodium chabaudi*

**DOI:** 10.1515/biol-2022-0968

**Published:** 2024-10-23

**Authors:** Mutee Murshed, Jameel Al-Tamimi, Khalid Elfaki Ibrahim, Saleh Al-Quraishy

**Affiliations:** Department of Zoology, College of Science, King Saud University, P.O. Box 2455, Riyadh 11451, Saudi Arabia

**Keywords:** glomerulonephritis, kidney cortex, tubular necrosis, biosynthesized

## Abstract

The study aimed to verify the pathogenic malarial kidney infections and histopathological pictures in mice infected with *Plasmodium chabaudi* using *Indigofera oblongifolia* leaf extract silver nanoparticles (IOLEAgNPs). Fifty healthy adult female mice C57BL/6 were used. Animals were divided into five groups, with each group of ten mice. The first control non-infected group was given distilled water for 7 days. The second group was orally given 50 mg/kg of IOLEAgNPs. The third, fourth, and fifth groups were injected intraperitoneally with 10^5^ parasitized erythrocytes of *P. chabaudi*. After 1 h, the fourth group received 50 mg/kg of IOLEAgNPs, while the fifth group orally received 10 mg/kg chloroquine phosphate. The histopathology of the kidney was studied by routine histology method with hematoxylin–eosin staining. The kidney revealed cerebral microvessel congestion, hemorrhages, and necrosis. Cast formation, glomerulonephritis, tubular necrosis, and congestion were observed in the kidney cortex. Consequently, the targeted medical IOLEAgNPs reduced this degeneration impact on renal tissue. Proven that plant-source synthesized IOLEAgNPs play a preventive role as antimalarial agents in female mice infected with *P. chabaudi.*

## 1 Introduction

*Plasmodium* parasites are responsible for the infectious disease known as malaria, which is spread to humans through the bites of female Anopheles mosquitoes that are infected with the disease while they are feeding on blood [1]. *Plasmodium falciparum* and *Plasmodium vivax* are the primary human species responsible for malaria cases and fatalities. The increased rates of morbidity and mortality caused by malaria are mostly attributed to the development of parasite resistance as well as a decline in the effectiveness of antimalarial medications and their derivatives, such as artemisinin and chloroquine [2]. In Myanmar’s Greater Mekong Sub Region, there have been reports of developing resistance to artemisinin combination therapy and experiencing a decline in its effectiveness [3]. An increase in the inhibitory concentration of 50%, phenotypic modifications of dormant form, faster growth following viable of latent form, and a mutation in the pfatpase6 gene were observed in *P. falciparum* that had been subjected to repeated artemisinin *in vitro*, according to the findings of the research conducted by Maslachah et al. [4]. Serious malarial pathogenesis is linked to the presence of infected red blood cell cytoadherence in endothelial cells, which results in the microvascular sequestration of parasites and the blockage of microvascular flow in important organs [5]. Pyelonephritis, often known as kidney infections, presents substantial worldwide health issues, impacting millions of people each year [6]. The complex structure of the kidney, which includes nephrons, blood arteries, and renal tubules, makes it vulnerable to several types of diseases, such as microbial invasion [7]. Notably, the kidney is an important organ that controls blood pressure, physiological fluid volume, acid–base balance, and the creation and release of specific hormones [8]. The histo-physiological changes in renal function were regarded as a detector for histological studies [9,10]. The literature extensively covered research on renal morphology and histology, which played a pivotal role in assessing the effects of innovative medicinal plants on disorders with nephrotoxic impact [11,12]. Natural medicines have received significant attention for their ability to fight infectious illnesses while minimising the possibility of resistance development [13]. *Indigofera oblgofola*, a traditional medicinal herb renowned for its antibacterial qualities, has shown potential in preclinical investigations [14]. Nevertheless, there is currently a dearth of complete knowledge about its therapeutic effectiveness in treating kidney infections [15]. Silver nanoparticles (AgNPs) have antimalarial properties [16]. Moreover, the antagonistic role of AgNPs synthesized by *I. oblongifolia* leaves extract (IOLEAgNPs) was demonstrated to evaluate its role in improving pathological alterations in spleen of mice during parasite infection with *Plasmodium chabaudi* [17]. Histomorphometric analysis is a very effective method used in biomedical research to get important understanding of the structural alterations that occur in tissues under diseased circumstances and in response to therapeutic treatments [18]. Histomorphometry is a method that measures factors including cellular density, tissue integrity, and inflammatory infiltrates to offer a precise evaluation of therapy effects [11,19].

The purpose of the study is to determine the therapeutic effect of *I. oblongifolia* leaf extracts utilized with AgNPs on infected kidneys using histomorphometry analyses. We will use standardized histology methods and modern imaging modalities to carefully examine the structural changes in renal tissue after administering IOLEAgNPs. In addition, we will investigate the possible processes that contribute to its anti-*Plasmodium chabaudi*-induced properties and its capacity to reduce inflammatory reactions in the kidney.

## 2 Materials and methods

### 2.1 Mice, parasites, and biosynthesized AgNPs used in the study

Fifty female C57BL/6 mice aged 9 ± 2 weeks (weighing 22 ± 3 g) were provided by the King Faisal Hospital Research Unit (Riyadh, Saudi Arabia). *P. chabaudi* strain was obtained from the parasitology Laboratory at the College of Science, King Saud University. *P. chabaudi* cryopreserved parasitic have been passed to uninfected mice (1 time). A phosphate buffer with a volume of 100 μL, which included 10^5^ erythrocytes that had been parasitized by *P. chabaudi*, was administered intraperitoneally (i.p.) from the mouse.

After preparing the extract according to the method [20]. In brief, the powdered leaves were stirred in methanol for 24 h. The extract was filtered using the Whatman No. 1 filter paper. Part of this filtrate (5 mL) was used for the preparation of biosynthesized AgNPs and the other part was evaporated using a vacuum evaporator (IKA, Germany). In distilled water, the residues were dissolved and deposited at −20°C before usage.

The procedure for the preparation of nanoparticles was followed by Naidu et al. [21]. Briefly, IOLE (5 mL) was applied to 8 × 10^−3^ M silver nitrate in 45 mL methanol, and then, the mixture obtained was held at 50°C for 60 min before it was transformed into a dark brownish color suggesting the formation of AgNPs in solution.


**Ethical approval:** The research related to animal use has been complied with all the relevant national regulations and institutional policies for the care and use of animals, and has been approved by the Animal Ethics Committee at King Saud University, Saudi Arabia (certificate number No. KSU-SE-21-86).

### 2.2 *In vivo* in the mice

Animals were divided into five groups. Each group consisted of ten mice. The first control non-infected group was given distilled water for 7 days orally. The second group was orally given 50 mg/kg of IOLEAgNPs 7 days daily. The third, fourth, and fifth groups were injected intraperitoneally with 10^5^ parasitized erythrocytes of *P. chabaudi*. After 1 h, the fourth group received 50 mg/kg of IOLEAgNPs daily for 7 days [17], while the fifth group orally received 10 mg/kg chloroquine phosphate (Sigma-Aldrich, St. Louis, MO) daily for 4 days [22]. On day 7 p.i., all animals were slain utilizing carbon dioxide CO_2_ asphyxiation and then dissected for collection of samples. The histopathology of the kidney was studied by routine histology method with hematoxylin–eosin (H&E) staining.

### 2.3 Histological assessment

#### 2.3.1 Collection of samples

All mice were sacrificed after CO_2_ asphyxiation on day 7 post-infection and then dissected to collect blood and tissues. Blood was collected from the heart and stored in a heparin-salted tube separated into two parts, one part for whole blood and the other part as a plasma separator. The plasma was then kept at −20°C until use. For the histological study, the kidney was isolated and divided into pieces. The small pieces were fixed in neutral buffered formalin (10%) for histopathological examination.

### 2.4 Kidney histopathology

The lobes of the left kidney from the control and treatment groups were fixed in 10% neutral buffered formalin for 24 h at room temperature. Fixed organs were embedded in paraffin, sectioned (4–5 μm), and stained with H&E routine protocols. The sections were then stained with H&E.

Sections were examined microscopically and changes were recorded using a standard non-linear semi-quantitative scoring system using a scale from 0 to 5 adapted from [23]. Significant findings were scored 0 (where no change was detectable), 1 when the least amount of change was detectable by light microscopy (usually <10% of tissue affected), 2 when the change was readily detected but not a major feature (<20%), 3 when the change was more extensive and might be expected to correlate with changes in organ weight or function, 4 when up to 75% of tissue was affected by the change, and 5 when the whole tissue was affected by a change which was likely to be functionally relevant. Organs from the control group were always compared with those from the treatment groups. The percentage of vessels in each organ containing infected red blood cells iRBC was determined from 100 vessels. In brief, the following steps were performed.

Kidneys were fixed immediately in 10% neutral buffered formalin, processed for light microscopy through a graded series of alcohol (100, 96, 90, 80, 70%), cleared in xylene, and embedded in paraffin wax to get paraffin sections that stained with Hematoxylin & Eosin (H & E).

Sections were stained with hematoxylin for 5–15 min to stain the nuclei. Then, sections were washed in tap water for 2 min until sections turned blue (bluing). Differentiation of sections in 70% ethanol containing 1% hydrochloric acid (HCl) for 1–5 s was done. This removed the excess dye and allowed nuclear details to emerge. After that, sections were washed for 3–5 min in tap water until blue. Sections were inserted in ammonia water-one dip (gives a blue color to the nuclei). The pieces were then rinsed in tap water. Sections were stained in eosin solution for 1–2 min to stain cytoplasm. Thereafter, sections were dehydrated in an ascending series of ethanol concentrations (90, 96, 100%) and cleared in xylene. Finally, sections were mounted in Dibutylphthalate Polystyrene Xylene DPX and covered by a coverslip. Stained sections were imaged using a light microscope (Leica, Wetzlar, Germany).

After being stored in 10% neutral buffered formalin, the organ specimen was wrapped in paraffin. H&E staining and processing of the 6 μm paraffin sections followed the protocol outlined by Bancroft and Layton [24].

To measure several morphological characteristics of the different investigated organs, such as alveolar wall thickness, the degree of necrosis intensity, and any structural abnormalities, we use the image analysis software ImageJ. We assess potential alterations in the histoarchitectural composition brought by *P. chabaudi* infection. The data gathered over five experimental groups were analyzed to ascertain the therapeutic impact of *I. oblgofola* AgNPs after infection. Using accepted statistical techniques, the means (*M*) and standard error (SE) of each set of data are determined [25,26]. After establishing the test’s significance (*P*-value) at the 5 or 1% level, an analysis of variance (ANOVA) is utilized to assess the interaction effects in more detail. Throughout the whole statistical analysis, the program SPSS for Windows, version 10.0, is utilized.

### 2.5 Statistical analysis

The data were analyzed with a one-way ANOVA and presented as the mean and standard deviation of three replications. The criterion of significance was fixed at *P* ≤ 0.05.

## 3 Results

Initially, the present study was designated to explore the therapeutic impact of *I. oblgofola* AgNPs on severe inflammatory signs that result from *P. chabaudi* infection in kidney.

### 3.1 Histomorphometric observations of kidney tissue

The current examination of the histological section of the kidney tissue exhibited a good histoarchitecture of renal structures by applying *I. oblgofola* AgNPs on the normal kidney. On the other hand, there was chronic nephrotic degeneration due to the infection of *P. chabaudi*.

The present investigation was based on five groups: the control group, the *I. oblgofola* AgNP group, the *P. chabaudi* infected group, the treated group by *I. oblgofola* AgNPs after infection, and another treated group by Chloroquine CQ10 mg/kg after infection.

#### 3.1.1 Control group

**Figure 1:**
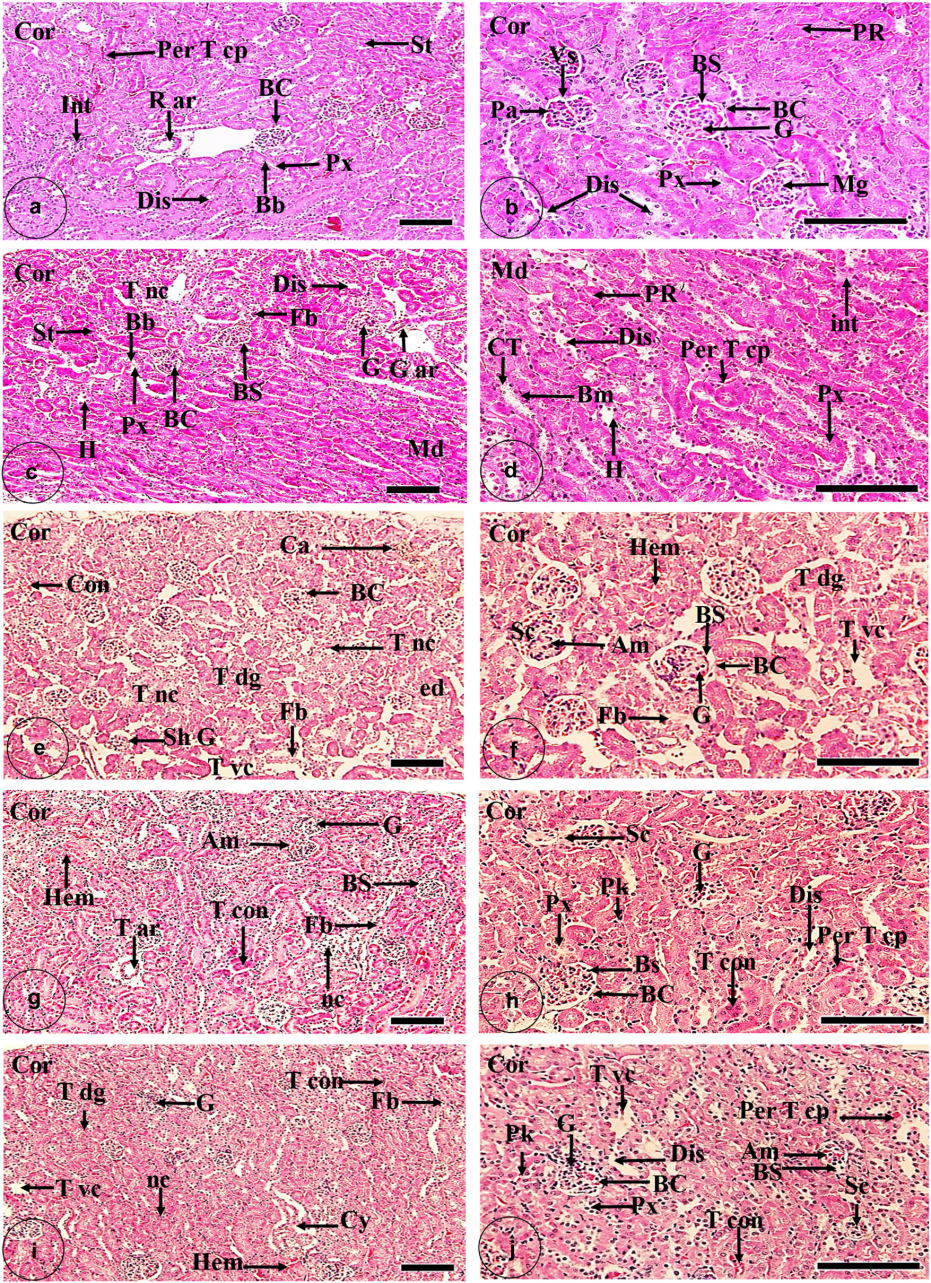
Bar charts showing the morphometric analysis of histopathological parameters of kidney tissue which (a) bowman’s capsule diameter, (b) glomerulus diameter, (c) diameter of bowman’s space, (d) diameter of the proximal tubule, (e) diameter of the distal tubule, (f) glomerulus sclerosis intensity, (g) amyloid deposition intensity, (h) hemorrhage intensity, (i) fibrosis intensity, and (j) tubular necrosis intensity; among five groups: control; IOLEAgNPs group; infected group by *P. chabaudi*, infected group by *P. chabaudi* and 50 mg/kg of IOLEAgNPs; and the administrated group by *P. chabaudi* and CQ10 mg/kg. Values are represented as mean ± standard deviation and *n* = 10 animals. Means within the same parameter and not sharing a common superscript symbol(s) differ significantly at *p* < 0.05, and values that are recorded with a non-significant difference (n.s.).

The gross landmarks of the normal kidney were the outer granular cortical region and inner tubular medullary area (Figure 1a, c and d). The functional unit of the kidney was the nephron which mainly constituted from renal capsule and complicated tubular system, whereas the renal capsule occupied most of the cortical space composed of the bowman’s capsule that was infiltrated by the cellular mass of mesangial cells that was terminated with glomerulus and surrounded by bowman’s space. The complex tubular network was constituted of the proximal convoluted tubule, pars recta, distal convoluted tubule, and collecting tubules. Both the renal capsule and tubular system were penetrated by some blood vessels such as the renal artery, peritubular capillaries, and interstitium regions (Figure 1a and b).

#### 3.1.2 *I. oblgofola* AgNP group

**Figure 2:**
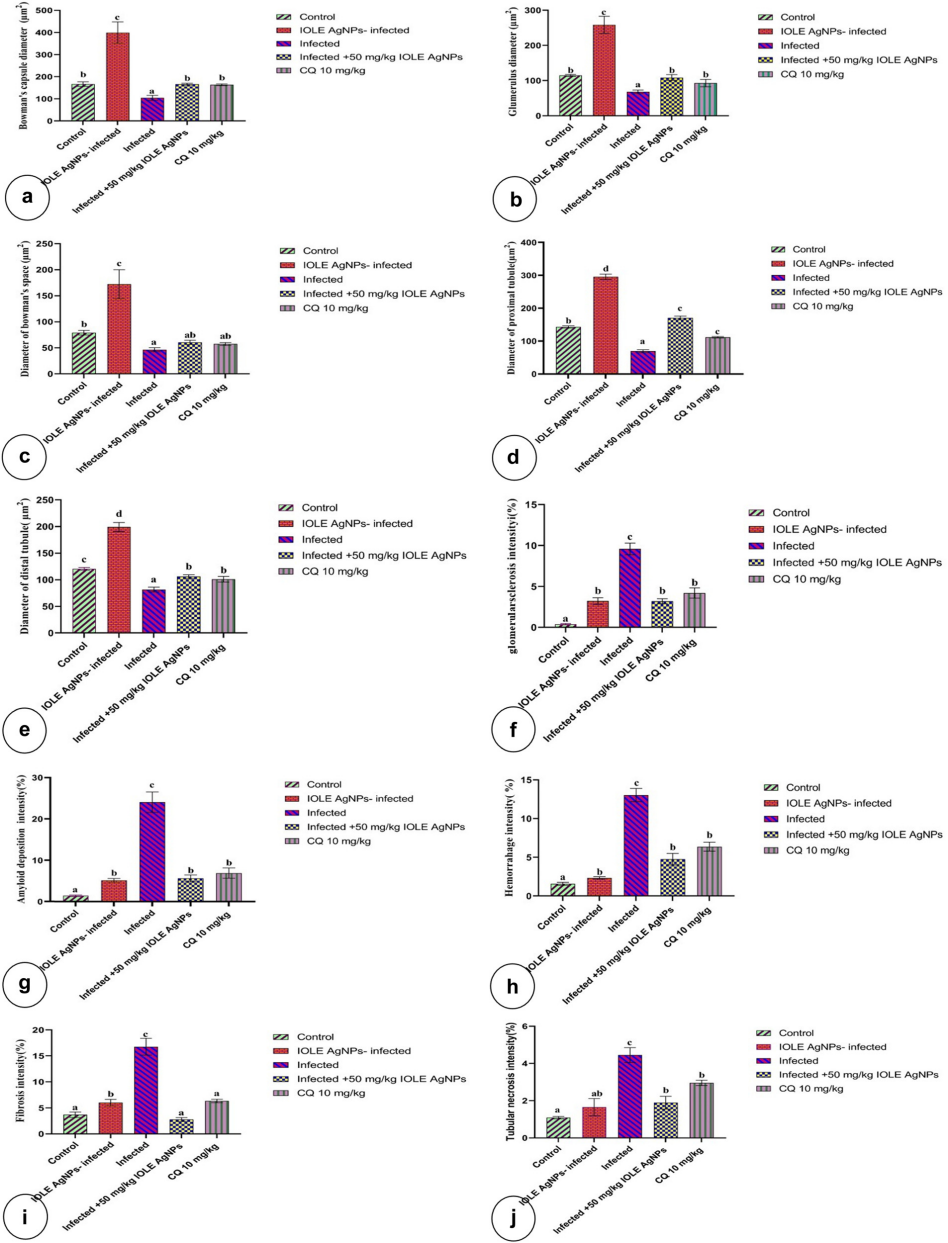
(a and b) Photomicrograph of the histological section of a control kidney showing the cortical area (Cor) comprised of a bowman’s capsule (Bc) that consisted of an internal glomerulus (G) surrounded by a bowman’s space (Bs). The mesangium (Mg) represents the internal component of the glomerulus. The outer layer of the glomerulus is called the visceral layer (Vs) and the inner layer of the bowman’s capsule is called the partial layer (Pa). The cortical stroma (St) is composed of proximal convoluted tubules (Px) with BB and distal convoluted tubules (Dis). Also, it includes the renal artery (R ar), pars recta (PR), peritubular capillaries (Per T cp), and interstitium (int) (H&E X, a: 100 µm and b: 400 µm). (c and d) Photomicrograph of histological sections of kidney tissue of the administrated group by IOLEAgNPs showing the cortical area (Cor) comprised a bowman’s capsule (Bc) that consisted of an internal glomerulus (G) surrounded by a bowman’s space (Bs). The cortical stroma (St) is composed of proximal convoluted tubules (Px) with BB and distal convoluted tubules (Dis). Also, it includes the glomerular artery (G ar) and exhibited some tubular necrosis (T NC), besides some fibrotic lesions (Fb). On the other hand, the medullary region (Md) composed of Henle loop (H), pars recta (PR), peritubular capillaries (Per T cp), interstitium (int), and collecting tubule (CT) with a significant basement membrane (Bm) (H&E X, c: 100 µm and d: 400 µm). (e and f) Photomicrograph of histological sections of kidney tissue of the infected group by *P. chabaudi* showing that the cortical area (Cor) comprised a bowman’s capsule (Bc) that consisted of an internal glomerulus (G) surrounded by a bowman’s space (Bs). Some histopathological features appeared in this section such as tubular necrosis (T nc), cast (Ca), tubular degeneration (T dg), congestion (Con), edema (ed), fibrosis (Fb), shrinkage of the glomerulus (Sh G), hemorrhage (Hem), glomerulosclerosis (Sc), amyloid deposition (Am), and tubular vacuolation (T vc) (H&E X, e: 100 µm and f: 400 µm). (g and h) Photomicrograph of histological sections of kidney tissue of the infected group by *P. chabaudi* and 50 mg/kg of IOLEAgNPs showing the cortical area (Cor) comprised a bowman’s capsule (Bc) that consisted of an internal glomerulus (G) surrounded by a bowman’s space (Bs). Reappearance of tubular structures such as proximal convoluted tubules (Px) and distal convoluted tubules (Dis). Also, it includes the tubular artery (T ar) and peritubular capillaries (Per T cp) and exhibited some necrosis (nc) with pyknotic cells (Pk), besides some fibrotic lesion (Fb). Also, some histopathological signs rested such as tubular congestion (T con), glomerulosclerosis (Sc), hemorrhage (Hem), and amyloid deposition (H&E X, g: 100 µm and h: 400 µm). (i and j) Photomicrograph of histological sections of kidney tissue of the administrated group by *P. chabaudi* and CQ10 showing that the cortical area (Cor) comprised a bowman’s capsule (Bc) that consisted of an internal glomerulus (G) surrounded by a bowman’s space (Bs). Reappearance of tubular structures such as proximal convoluted tubules (Px) and distal convoluted tubules (Dis). Also, it includes peritubular capillaries (Per T cp) and exhibited some necrosis (nc) with pyknotic cells (Pk), besides some fibrotic lesions (Fb). Also, some histopathological signs rested such as tubular congestion (T con), tubular degeneration (T dg), tubular vacuolation (T vc), cyst (Cy), glomerulosclerosis (Sc), hemorrhage (Hem), and amyloid deposition (H&E X, i: 100 µm and j: 400 µm).

The administration of *I. oblgofola* as a traditional medical plant besides AgNPs as an anti-inflammatory and anti-carcinogenic substance led to a wide improvement in the renal histoarchitecture in general. This meaningful refinement appeared in the significant increase (*P* < 0.001) of the diameter of the bowman’s capsule, bowman’s space, glomerulus, and proximal and distal tubule more than control one’s (Figure 2a–e). Also, the proximal convoluted tubules appeared with well-standing brushing borders (BB). The collecting tubules and the Henle loop exhibited well-rounded nuclei basement membrane (Figure 1c and d). Conversely, the renal stroma possessed some necrotic tubules (1.64 ± 0.18%) more than normal stroma as well as little intensity of fibrosis (6.02 ± 0.26%) more than the standard one (Figure 1i and j).

#### 3.1.3 *P. chabaudi* infection group

The nephrotic damage appeared as pathological signs on the renal constituents like glomerulus and different types of tubules after administration of *P. chabaudi* infection. Multiple histopathological signs were documented in the mouse kidney in this group such as; casts and congestion of the interstitium. As much as tubular degeneration and vacuolation diffused over the whole tissue. The infected glomerulus demonstrated severe shrinkage (Figure 1e and f). Frequently, edema was exhibited in the cortical region. Renal capsules in general exposed to hard shrinkage whereas the bowman’s capsule diameter (104.56 ± 4.55 µm^2^), bowman’s space diameter (46.42 ± 1.53 µm^2^), and glomerulus diameter (68.12 ± 2.05 µm^2^) reduced less than the ordinary capsule. For this reason, glomerular sclerosis (9.58 ± 0.29%) and amyloid deposition (24.05 ± 1.00%) increase more than in the control glomerulus (Figure 2a–c). Comparatively, the tubular system revealed tough degeneration that was demonstrated in the reduction of the diameter of the proximal and distal tubules less than the regulatory ones. This group had significantly higher tubular necrosis (4.45 ± 0.16%) compared to other groups (Figure 2j). Furthermore, the severity of fibrosis and bleeding in this group’s renal stroma was higher than in the control stroma.

#### 3.1.4 Treated group by *I. oblgofola* AgNPs after infection

By applying *I. oblgofola* AgNPs on the infected rats, the nephrotic degeneration expressed general improvement in all histological features more than those of the infected ones and also a reduction in all histopathological signs less than that not treated. It was observed that glomerular sclerosis, amyloid deposition, hemorrhage, fibrosis, and tubular necrosis registered a significant reduction less than in the nontreated groups (Figure 2e–j). The renal capsule restored its relatively normal size after treatment significantly by raising the diameter of the bowman’s capsule, space, and glomerulus. Phenomena of tubular degeneration disappeared after increasing the diameter of the proximal and distal tubules. However, the renal stroma still had tubular congestion and necrosis over the whole tissue.

#### 3.1.5 Treated group by CQ10 mg/kg after infection

Another traditional treatment that was applied to the infected rats with *P. chabaudi* was CQ10 mg/kg. It showed a slight refinement to the degeneration status of the infection by decreasing the glomerular sclerosis intensity in addition to hemorrhage, amyloid deposition, fibrosis, and tubular necrosis intensity significantly (*P* < 0.000) less than in the diseased rats (Figure 2e–j). Conversely, the current investigation clarified that there was a non-significant variation between the traditional drug CQ10 mg/kg and the novel treatment *I. oblgofola* AgNPs in all morphometric measurements that were taken during the phase of the treatment (Figure 1i and j).

**Figure 3:**
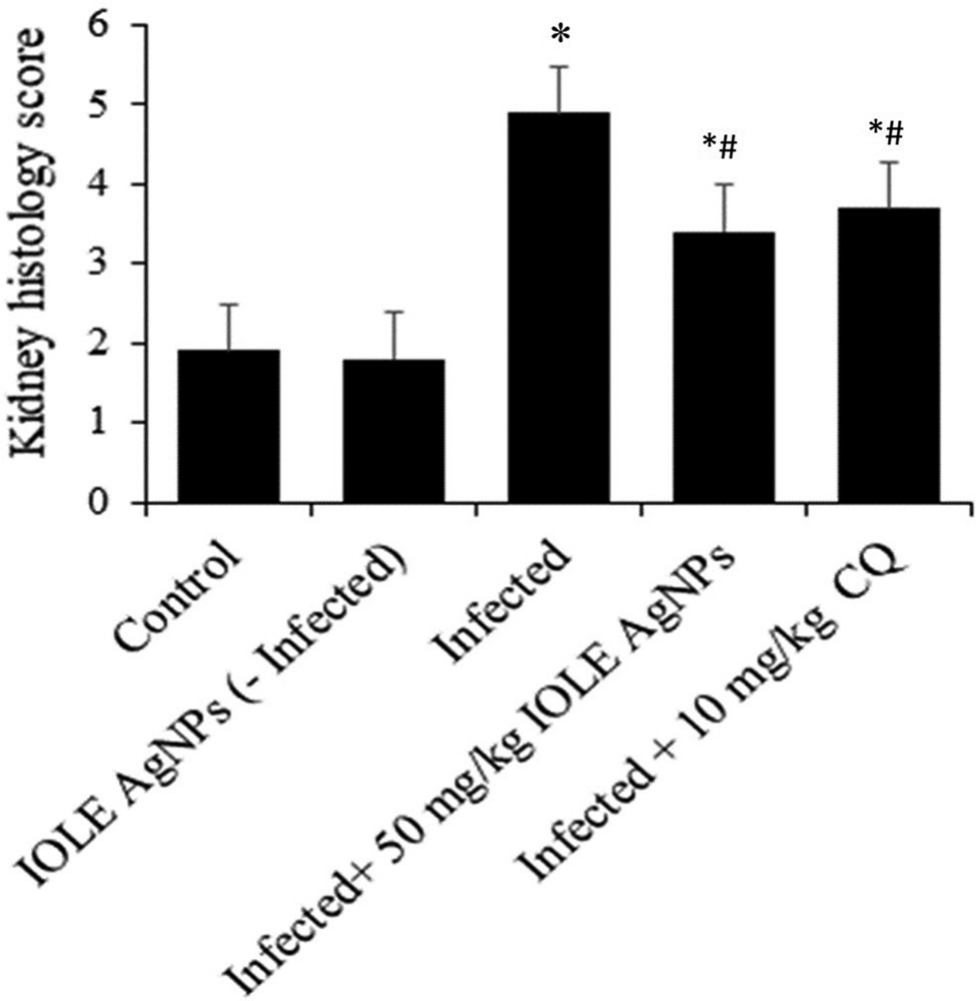
IOLEAgNPs induced score in the kidney function of mice infected with *P. chabaudi*. * and # are significant at *p*  <  0.01 against control and infected groups, respectively.

The score for kidney histology was calculated to be around fivefold when compared to the control group. However, when mice were treated with IOLEAgNPs, the score reduced to approximately thrice (Figure 3).

**Figure 4:**
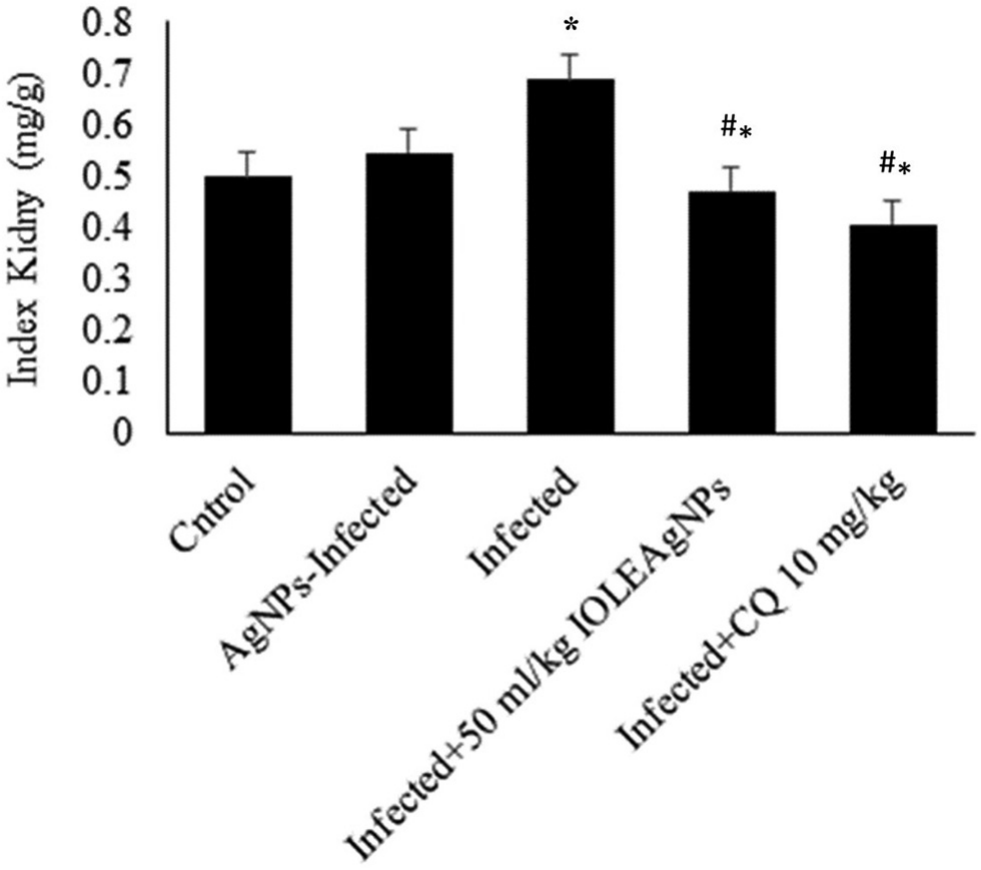
IOLEAgNPs induced changes in the kidney function index of mice infected with *P. chabaudi*. * and # are significant at *p*  <  0.01 against control and infected groups, respectively.

The infection induced hepatomegaly, as demonstrated by the increased liver index in mice on day 7 after infection with *P. chabaudi* (Figure 4). IOLEAgNPs could significantly decrease the liver index by about one time nearly similar to that induced by the CQ-treated group.

## 4 Discussion

Eventually, the current investigation was based on the study of the *I. oblgofola* AgNPs’ influence on the normal mouse and also its therapeutic impact on infected rats with the malaria parasite. By application of AgNPs with their anti-inflammatory activity, anti-microbial, and anti-carcinogenic properties in combination with *I. oblgofola* as a bioavailability medical plant on the normal mouse, it was expected that the histological and physiological status will be amended with a high degree.

First, a recent study revealed some improvement in the histoarchitecture of the kidney structure besides, little atrophic signs were expressed due to the filtration of nanoparticles through the kidney tubules. This investigation recorded hypertrophy in the renal capsule in general more than all studied groups, in addition to significant (*P* < 0.001) expansion in the diameter of both proximal and distal tubules. A study [27] referred to the glomerular and tubular hypertrophy to the filtration of nanoparticles through nephrotic components leading to slight cytotoxicity. The data analysis showed that animals administered with nanoparticles had significantly higher levels of bleeding, amyloid deposition, fibrotic lesions, and tubular necrosis compared to control mice. A study [28] recorded renal toxicity in albino rats after the repetition of oral administration of AgNPs and explained the direct impact of nanoparticles on cellular construction through raising reactive oxygen species generation leading to necrotic and fibrotic alterations. On the other hand, many research confirmed the treatment influence of *I. oblgofola* on the prevention the inducing renal and hepatic toxicity as it enjoys anti-oxidative, anti-inflammatory, antifibrotic, and antiapoptotic properties [29]. The recent analysis agrees with the previous explanation due to the combination of AgNPs and *I. oblgofola* relieving the stressful impact on the cellular oxidative species.

The experimental group of infected parasite rats displayed significant (*P* < 0.000) histopathological signs that included hemorrhage, amyloid deposition, more fibrotic lesions, higher tubular necrosis intensity, and also higher glomerular sclerosis than other experimental groups. As malaria was considered the most human popular parasite that was usually transmitted through the *Plasmodium* parasite; therefore, they were considered chronic renal injury related to malaria infection [30]. A study [31] described the nephrotic syndrome as a direct effect of *Plasmodium* on the renal cellular structures. Also, he manifested acute interstitial nephrites, tubular necrosis, and various inflammatory symptoms as detectable signs of malaria in the kidney. The current clinical manifestations demonstrated severe hemorrhage, hemosiderin intensity, tubular degeneration, and vacuolation. A study [32] recorded that kidney involvement in malaria exhibited urinary cast formation similar to the existing analysis in this research.

The treated experimental rats with *I. oblgofola* AgNPs showed refinement symptoms to a severe nephrotic syndrome caused by *Plasmodium*. The present data revealed that the *I. oblgofola* treatment had well imprint on acute kidney injury of malaria [11,29]. *I. oblgofola* has been used for its analgesic and anti-inflammatory properties to alleviate pain. This medical plant yields unique compounds called indigin and indigoferic acid, which are the fatty acid ester of *p*-hydroxy (*E*)-cinnamic acid and alkylated xanthenes, respectively. The plant was also found to contain 3-hydroxybenzoic acid and β-sitosterol. Hepatocytes are protected from carbon tetrachloride by *I. oblongifolia*-induced hepatotoxicity by inhibiting the oxidative stress-induced oxidation of proteins, deoxyribonucleic acid, and membrane lipids.

Finally, the experimentally infected rats that were injected with another treatment CQ10 mg/kg showed a low rate of improvement after infection with *Plasmodium* due to higher levels of hemorrhage, amyloid deposition, glomerular sclerosis, hemosiderin intensity, fibrotic lesions, and tubular necrosis more than in the previous group of *I. oblgofola* treatment. The recent observation in this group of experimental rats also recorded that the pyknosis phase was considered the primary process of necrosis which indicated the little efficiency of this treatment. A study [33] discussed the pyknosis status due to the loss of ribosomal ribonucleic acid from the endoplasmic reticulum causes pyknosis, which develops in the nuclei of the renal glomeruli as a result of death-inducing factors represented by cytochrome *C* released from mitochondria. The chromatin of nuclei becomes condenser in the dark mass form, which represents one type of necrosis. Correspondingly, there was a refinement in the general histological structure after the supplementation with CQ10 mg/kg besides recording a significantly higher level in the renal capsule diameter than the renal capsule of the infected groups and displayed lower intensity of glomerular sclerosis than the diseased rats. A study [34] referred this improvement in nephrotic syndrome to lower oxidative stress, enhance mitochondrial function, and lessen unfavorable cardiovascular events after CQ10 mg/kg supplementation for cardiovascular disease.

## 5 Conclusion

In summary, the current investigation proved that the efficiency of *I. oblgofola* treatment was more obvious than CQ10 mg/kg due to low intensities of all morphometric histopathological signs such as hemorrhage, amyloid deposition, glomerular sclerosis, hemosiderin intensity, fibrotic lesions, and tubular necrosis in the injected rats with *I. oblgofola* less than CQ10 mg/kg rats. Mice treatment with *I. oblgofola* showed a quick recovery in the diameter of the renal capsule and removal of it from amyloid accumulation. The results of this study have important significance for the advancement of alternative treatment approaches for kidney infections.
